# Improving Heart Healthy Lifestyles Among Participants in a *Salud Para Su Corazón* Promotores Model: The Mexican Pilot Study, 2009–2012

**DOI:** 10.5888/pcd12.140292

**Published:** 2015-03-12

**Authors:** Héctor Balcázar, Ana Cecilia Fernández-Gaxiola, Ana Bertha Pérez-Lizaur, Rosa Adriana Peyron, Carma Ayala

**Affiliations:** Author Affiliations: Ana Cecilia Fernández-Gaxiola, Ana Bertha Pérez-Lizaur, Rosa Adriana Peyron, Universidad Iberoamericana Ciudad de México, México City, México; Carma Ayala, Centers for Disease Control and Prevention, Atlanta, Georgia.

## Abstract

**Introduction:**

In Mexico, cardiovascular disease and its risk factors are growing problems and major public health concerns. The objective of this study was to implement cardiovascular health promotion and disease prevention activities of the *Salud para su Corazón* model in a high-risk, impoverished, urban community in Mexico City.

**Methods:**

We used a pretest–posttest (baseline to 12-week follow-up) design without a control group. Material from *Salud para su Corazón* was validated and delivered by promotores (community health workers) to community members from 6 geographic areas. Two validated, self-administered questionnaires that assessed participants’ knowledge and behaviors relating to heart health were administered. We used *t* tests and χ^2^ tests to evaluate pretest and posttest differences, by age group (≤60 and >60 years), for participants’ 3 heart-healthy habits, 3 types of physical activity, performance skills, and anthropometric and clinical measurements.

**Results:**

A total of 452 (82%) adult participants completed the program. Heart-healthy habits from pretest to posttest varied by age group. “Taking action” to modify lifestyle behaviors increased among adults aged 60 or younger from 31.5% to 63.0% (*P* < .001) and among adults older than 60 from 30.0% to 45.0% (*P* < .001). Positive responses for cholesterol and fat consumption reduction were seen among participants 60 or younger (*P* = .03). Among those older than 60, salt reduction and weight control increased (*P* = .008). Mean blood glucose concentration among adults older than 60 decreased postintervention (*P* = .03).

**Conclusion:**

Significant improvements in some heart-healthy habits were seen among adult participants. The model has potential to improve heart-healthy habits and facilitate behavioral change among high-risk adults.

## Introduction

In Mexico, cardiovascular disease (CVD) and its risk factors are growing problems and major public health concerns (1–4). According to a report by the World Health Organization, in 2008, the age-standardized death rate per 100,000 for CVD and diabetes was 217 for Mexican adults compared with 137 in the United States (2). Furthermore, in 2008 the first and second leading causes of death in Mexico were diabetes (70.8) and ischemic heart disease (55.8), followed by cerebrovascular events (28.3) (age-standardized death rate per 100,000 population) (3). The most prevalent CVD risk factors for men and women in Mexico are obesity (26.7% and 38.4%), smoking (30.0% and 18.0%), hypertension (27.4% and 21.5%), and diabetes (13.2% and 14.9%) (1,4,5). As with many countries, Mexico’s CVD burden and risk factors are not evenly distributed among all social and economic sectors of the society (1–3,5–7). Economic and social influences (eg, educational, cultural, and environmental factors) are powerful predictors of CVD and risk factors in Mexico (7–9).

Social influences limit the capacity to respond effectively to address the high burden of CVD in Mexico (7). People living just above the poverty threshold in Mexico may have limited access to primary health care because they do not meet the qualifications for government-provided programs (7). Educational, cultural, and environmental factors may lead to difficulties in understanding health information and recommendations to promote health and healthy lifestyles (6,7). Lack of access to health care among the older poor can lead to undiagnosed or poorly controlled risk factors for CVD such as hypercholesterolemia, hypertension, and diabetes (8–10).

The burden of CVD and its risk factors can be reduced by delivering community-based programs that promote healthy lifestyle behaviors that lead to healthy diets, weight management, and a physically active lifestyle (11–13). We adapted and implemented a community-based program developed for US Hispanic populations called *Salud para su Corazón* to a Mexican context. The program aims to increase knowledge about CVD risk factors and heart-healthy behaviors among Hispanic populations and has been widely and successfully implemented in the United States (14–20). The purpose of this article is to describe the results of the Mexican *Salud para su Corazón* model pilot study for promoting healthy behaviors and reducing CVD risk factors by incorporating promotores de salud (community health workers) in a high-risk, poor, urban community in Mexico City, District Federal (DF).

## Methods

### Participants

Participants were included in the study if they were aged 18 years or older, living in the community at the time of the study, planning to stay for the next 12 weeks, and willing to sign an informed consent form. The refusal rate for eligible participants was less than 3%; reasons given included not having time and family reasons. No additional exclusionary criterion was included. A total of 550 adults participated. Of those, 452 (82%) completed the pretest (baseline) and posttest (12-week postintervention follow-up) surveys and the anthropometric and clinical measurements. Ninety-eight participants (18%) did not complete the full program. Reasons for withdrawal were searching for or having started a new job and no longer being able to attend the sessions, no longer having time to attend the sessions, or having to take care of someone (child or adult). Most children and grandchildren in the community were at school, allowing participants to have time to attend the sessions.

Recruitment of promotores was done through community networks of the collaborative Department of Health (DH)–Universidad Iberoamericana (DH-UIA) and the target community. A total of 22 promotores were recruited and agreed to participate in the training, and the 12-week intervention *Salud para su Corazón* training was conducted by a lead promotora from El Paso, Texas. All promotores completed the 5-day training workshop and received the *Su Corazón Su Vida* curriculum materials (16–20). The team training for the promotores was conducted by researchers from the DH-UIA and the University of Texas, School of Public Health, El Paso Regional Campus (UTSPH-El Paso) (21). The promotores were assigned to 9 sites in different geographic areas of the community; working in pairs, they delivered 9 *Salud para su Corazón* lessons (16–18). Promotores started working in 2 health clinics; however, most group workshops were held at participants’ homes. Promotores were responsible for recruiting community participants primarily through outreach to neighborhood and community sites near the health clinics, and they formed groups with approximately 20 participants.

### Target community

In 2008, the DH-UIA in Mexico City, DF, in collaboration with the UTSPH-El Paso, initiated a promotores program as part of the university’s commitment to social responsibility within the community (21). The partnership focused on CVD risk factors in the community that were consistent with the *Salud para su Corazón* materials and programs to allow community organizations to pilot test the *Salud para su Corazón* model. The community of Santa Fe was selected because it is a tight-knit community in Mexico City, DF, with significant health risk (eg, people of low socioeconomic status [SES]). Community leaders (21) were invited to participate during an intensive week of well-defined and structured promotores training activities, following protocols similar to those previously implemented in other *Salud para su Corazón* programs in the United States (16–18).

### Intervention design

A community-based, pretest–posttest design without a control group was used as part of this pilot outreach program. A sample of 550 participants was examined at baseline (pretest) and given a 12-week behavior-change intervention. After the intervention, participants were re-examined and asked to complete the follow-up posttest survey (20,22). The study sample size was calculated to allow for detection of significant pretest and posttest differences from 18% to 24% (α = 0.05, sample power = 0.80). Randomization and use of a control community presented fiscal challenges and was not feasible. Conducting a randomized trial necessitated negotiating to identify control communities and finding resources that were not available at that time; furthermore, community leaders felt all participants should receive the intervention. The UIA-DH has a solid presence in the target community; it operates a community-based organization that offers varied social and development programs to surrounding neighborhoods.

Sessions were delivered at each site in group format once per week for approximately 2 hours, for a total of 12 weeks. At least once every 2 weeks, sessions were supervised by a team researcher who visited each site. *Salud para su Corazón* workshops were implemented over a 3-year period (2009–2012). After a session ended, a new group was formed and the same protocols for recruitment and intervention delivery were used to complete each of the 12-week interventions until the completion of all workshops.

The* Su Corazón su Vida* curriculum was adapted to the Mexican context and included 8 lessons (1 lesson per session). An extra ninth lesson on diabetes prevention and control, and the corresponding educational materials that accompany the lesson, were developed in collaboration with the UTSPH-El Paso team, because the topic was considered relevant in the curriculum for the target community. Identical questionnaires were used for the pretest and posttest to evaluate change related to the intervention.

### Measures

Pretest and posttest anthropometric measurements were taken by health care providers at the DH–UIA Nutrition Clinic. Anthropometric measurements of weight, height, and waist circumference were measured using standardized protocol in the DH-UIA Nutrition Clinic (23). Body mass index (BMI, kg/m^2^) was calculated using the World Health Organization reference values (24). Overweight was defined as a BMI of 25.0 to 29.9 and obesity was defined as a BMI of 30.0 or higher.

Clinical measures included blood pressure measurements and fasting glucose levels. Blood pressure was measured twice at each visit by health care providers in the dominant arm using the manual auscultation method with a mercury sphygmomanometer (American Diagnostic Corporation). Blood pressure was categorized as high blood pressure (ie, hypertension) if the participants had a systolic blood pressure of 140 mm Hg or higher or diastolic blood pressure of 90 mm Hg or higher on 2 separate readings (25). Standard classifications for fasting plasma glucose levels were used for diagnoses of prediabetes (100–125 mg/dl or 5.6–6.9 mmol/l) and diabetes (≥126 mg/dl or 7.7 mmol/l) (26).

Three validated self-administered questionnaires were used from previous *Salud para su Corazón* programs (12–14,20). A cardiovascular health knowledge survey and a program evaluation questionnaire contained closed-ended, curriculum-based questions (19). To assess behavioral changes the “My Habits” survey was used (12,18,20). The survey assessed participants’ heart-healthy behaviors and performance skills associated with reduction in salt or the consumption of foods with high sodium content (reduction of sodium intake) and cholesterol and fat consumption; weight control; and personal and family daily physical activity (eg, used stairs, got off bus and walked, walked, gardened). Additionally, participants recorded their personal physical activity (with or without family) by 3 exercise types: type A was 10 minutes, 3 times per day on some days of the week; type B was 30 minutes per day on some days of the week; and type C was 30 minutes per day, 3 or more times per week. Acceptance of the program was done using previous *Salud Para Su Corazón* protocols (14,16–18).

### Analyses

Participants were evaluated at baseline and after the 12-week intervention was completed (pretest and posttest). Frequencies of responses to each question were calculated on the basis of computed percentages and standard errors (SEs) or averages and standard deviations (SDs). We used *t* tests and χ^2^-tests to evaluate pretest and posttest differences for heart healthy habits, performance skills, and anthropometric and clinical measurements. Age categories were created on the basis of the mean and mode of the continuous age distribution (yielding 2 age groups with reasonable distribution of participants, representing mean values and ranging a span of 25-year difference for categories): aged 60 years or younger (mean age, 44.6 y) and older than 60 years (mean age, 70.4 y). The McNemar test was used to assess differences in proportions. Participants were stratified by socioeconomic risk factors (eg, health care access, access to healthy food) to examine differences between age groups (12,13,27).

## Results

### Demographic, anthropometric, and clinical measures

A total of 22 promotores participated; their mean age was 46.1 years, and 86% were women. All promotores reported they had access to health care services and had attended some school (Table 1).

**Table 1:** Socioeconomic Characteristics^a^ of Promotores and Adult Participants, *Salud para su Corazón* Mexican Pilot Study, 2009–2012

Characteristic	Promotores (N = 22)		Adult Participants (N = 452)				*P* Value^b^
			≤60 y (n = 279)		>60 y (n = 173)		
	n	% (SE)	N	% (SE)	N	% (SE)	
**Female**	19	86.3 (0.1)	157	56.3 (0)	76	43.7 (0)	.57
**Living situation**							
Lives with children/adolescents	7	31.8 (0)	189	67.7 (0.2)	56	32.3 (0.1)	.09
Lives with other adults	8	36.3 (0.1)	183	65.9 (0.2)	59	34.1 (0.1)	.03
Lives with older adults	5	22.7 (0.1)	109	38.9 (0.1)	106	61.1 (0)	.14
**Education level**							
Elementary school complete	2	9.2 (0)	58	20.5 (0)	31	17.9 (0.1)	.24
Secondary school complete	5	22.7 (0)	50	17.9 (0)	12	6.7 (0.1)	.04
High school complete	4	18.1 (0.1)	22	7.7 (0.1)	3	1.5 (0.2)	NA
Technical degree (2 y completed)		—	19	6.7 (0)	5	4.6 (0.1)	NA
Bachelor degree (4 y completed)	11	50.0 (0.1)	15	5.1 (0)	4	2.1 (0.1)	NA
Never attended school		—	2		15	8.7 (0)	NA
Not responded		—	114	40.8 (0.3)	102	58.5 (0.2)	.008
**Employed**	20	90.9 (0.1)	143	51.2 (0.1)	78	45.2 (0.2)	.04
**Access to health care services**	22	100.0 (0)	143	51.5 (0.1)	82	48.5 (0.2)	.10
**Takes care of another person**	2	9.0 (0.2)	143	51.5 (0.1)	82	48.5 (0)	.21
**Receives income**	17	77.2 (0.1)	143	51.3 (0.1)	78	45.7 (0)	.05
**Has free time**	20	90.9 (0.1)	143	51.5 (0)	82	48.5 (0)	.27

Abbreviations: NA, not applicable (sample size too small [<10] for reliable testing); SE, standard error.

a Mean (standard deviation) age in years of participants: promotores, 46.1 (13.5); participants aged 60 or younger, 44.6 (11.7); and participants older than 60, 70.4 (7.8).

b χ^2^ test comparing differences between adult participants by age group (>60 y and ≤60 y).

Of all participants, the mean age of those aged 60 years or younger was 44.6 years, and the mean age of those older than 60 was 70.4 years. Most participants aged 60 or younger were women (56.3%). The proportion of participants who completed secondary school was lower among adults older than 60 (6.7% vs 17.9%, *P* = .04) than for those aged 60 or younger. A lower proportion of adults older than 60 (45.2%) than adults 60 or younger (51.2%) were employed; therefore, a lower proportion of adults older than 60 (45.7%) received income than did adults 60 years or younger (51.3%, *P* = .05) (Table 1).

No significant differences were found between pretest and posttest anthropometric measurements among adults aged 60 or younger (Table 2). The posttest proportion of adults older than 60 classified as overweight (40.7%) was higher than those aged 60 or younger (30.0%; *P* = .045). Secondary analyses showed that adults who were classified as obese at baseline (pretest) were reclassified as overweight at posttest measurements, resulting in an increase in overweight estimates at the 12-week follow-up; adults who were classified as normal weight remained constant from baseline to follow-up (data not shown).

**Table 2:** Pretest (Baseline) and Posttest (12-Week Postintervention Follow-Up) Anthropometric and Clinical Measurements Among Adult Participants, by Age Group, *Salud para su Corazón* Mexican Pilot Study, 2009–2012

Measurement	N	Adults ≤60 y		Adults >60 y			*P* Value^a^
		Pretest	Posttest	N	Pretest	Posttest	
Mean height, (SD) cm	279	1.6 (0.7)	—	173	1.5 (0.1)	—	.29
Mean weight, (SD) kg	278	72.2 (16.3)	73.7 (15.3)	173	68.5 (13.1)	66.51 (16.1)	.30
Mean BMI, (SD) kg/m^2^	278	29.1 (5.6)	32.7 (11.6)	173	30.9 (5.1)	30.1 (5.4)	.26
Mean waist circumference, (SD) cm	242	94.6 (15.1)	92.6 (19.8)	158	99.4 (12.5)	94.7 (1.6)	.10
Fasting blood glucose, (SD) mg/dl	267	104.9 (37.4)	104.5 (39.0)^b^	160	129.9 (73.9)	116.7 (52.1)	.03
Mean systolic pressure, (SD) mm Hg	248	120.0 (15.5)	120.2 (17.0)^b^	157	126.0 (23.2)	141.0 (21.1)	.04
Mean diastolic pressure, (SD) mm Hg	247	79.4 (11.4)	78.0 (13.0)	158	72.9 (13.3)	77.2 (13.9)	.38
Overweight^c^, %	278	29.4	30.0	173	33.8	40.7	.045
Obese^c^, %	278	47.2	44.3	173	52.5	41.8	—
Alcohol intake, %	245	13.5	11	113	14.5	5.3	.02
Visit the doctor regularly, %	245	50.2	62.3	163	81.6	85.5	.04
Smoking in the family, %	279	44.1	—	163	60.5	—	NA
Family history of cardiovascular disease, %	279	61.9	—	145	55.8	—	NA
Family history of diabetes, %	279	71.8	—	145	65.8	—	NA
Family history of overweight /obesity, %	279	75.7	—	145	70.8	—	NA

Abbreviations: BMI, body mass index; NA, not applicable (sample size too small [<10] for reliable testing).

a χ^2^ test comparing differences of posttest responses between age groups (>60 and ≤60 years).

b *P* for difference between pretest and posttest within age group = .02; calculated using χ^2^ test.

c Overweight was defined as a BMI of 25.0–29.9, and obesity was defined as a BMI of 30.0 or higher.

Pretest mean glycemic values among adults older than 60 years were higher than among adults aged 60 years or younger (129.9 mg/dl and 104.9 mg/dl; *P* = .03) (Table 2). Posttest mean systolic blood pressure among adults older than 60 years was higher than among those aged 60 years or younger (140.9 mm Hg and 120.2 mm Hg; *P* = .02). Among those older than 60 years, the pre- to posttest comparison showed a decrease in the mean glycemic values (129.9 to 116.7; *P* = .03) but an increase for the mean systolic blood pressure (125.9 to 140.9; *P* = .04).

### Self-reported measures

**Figure 1:**
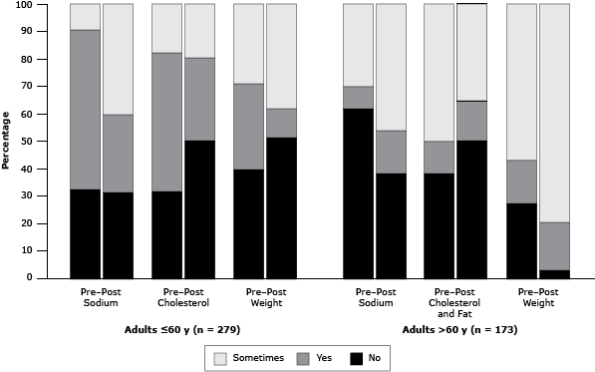
Distribution of responses for heart-healthy habits among adult participants at baseline (pretest) and 12-week postintervention follow-up (posttest) in the *Salud para su Corazón* Mexican pilot study. Responses were positive if the respondent answered yes for improved (less salt, saturated fat, and cholesterol consumption, more weight control or weight reduction) or “sometimes” improved on some days but not all the time. Negative responses if the respondent answered “no” meaning the positive health behavior is the same or decreasing (such as more or same amount of salt intake or cholesterol and fat consumption, less weight control or weight gain). **Table:** 

Comparing pretest to posttest responses among adults aged 60 or younger, positive responses for the reduction of cholesterol and fat consumption (*P* = .03) and weight control decreased (*P* = .04) (Figure 1). Among participants older than 60, posttest positive responses for reduction in sodium intake (*P* = .008) and weight control increased (*P* = .009) but decreased for cholesterol and fat consumption (*P* = .02), compared with the pretest responses.

Age Group/Habit	Pretest, %			*P* Value^a^	Posttest, %			*P* Value^a^
	Yes	No	Sometimes		Yes	No	Sometimes	
**Adults ≤60 y (n = 279)**								
Sodium intake	58.4	32.3	9.3	.001	28.3	31.2	40.5	<.001
Cholesterol and fat intake	50.5	31.5	17.9	.001	30.1	50.2	19.7	.001
Weight	31.2	39.8	29.0	.002	10.4	51.3	38.4	<.001
**Adults >60 y (n = 173)**								
Sodium intake	8.1	61.8	30.1	.001	15.6	38.2	46.2	.001
Cholesterol and fat intake	11.6	38.2	50.3	.24	14.5	50.3	35.3	.21
Weight	15.6	27.2	57.2	.38	17.3	2.9	79.8	.28

^a^ χ^2^ test comparing differences of posttest to pretest responses within each category.

**Figure 2:**
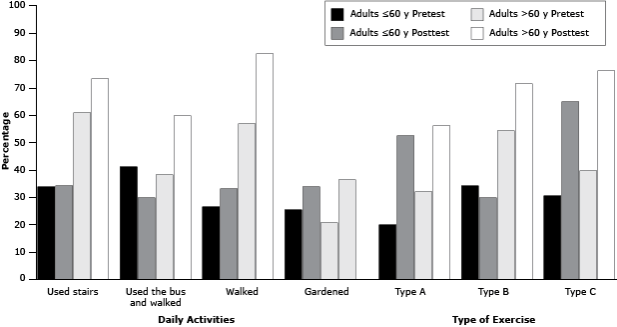
Percentage of positive responses about various physical activities among participants from baseline (pretest) to 12-week post-intervention follow-up (posttest) in the *Salud para su Corazón* Mexican pilot study. Physical activities were reported as percentage of time doing the following: using stairs, using the bus and walking, walking, gardening, or performing 3 types of exercise: type A (exercised 10 minutes, 3 times/day, some days of the week), type B (exercised 30 minutes/d, some days of the week), and type C (exercised 30 minutes/d, ≥3 times/week). **Table:** 

Among adults aged 60 or younger, the percentage of those who engaged in type A exercise (for 10 minutes, 3 times/day on some days of the week) increased from baseline to follow-up, but the change was not significant (Figure 2). The percentage of participants who engaged in type B exercise (30 minutes/d on some days of the week) decreased from pretest to posttest (34.2% to 29.7%, *P* = .04), and the percentage of adults aged 60 or younger who engaged in type C exercise (30 minutes, ≥3 times/week) increased from baseline to follow-up (30.6% to 65.0%, *P* < .001). Similarly, there were increases from pretest to posttest among those aged 60 or younger in the percentage of positive responses for those who walked or gardened (*P* = .30). However, there was a decrease from pretest to posttest in the percentage of positive responses for adults who reported type B exercise (30 min/d, some days of the week, *P* = .04) and among those who reported using a bus and walked (*P* = .02). Among adults older than 60, the percentage of positive responses for all 3 types of reported exercise increased from pretest to posttest (*P* < .001 for all) and increased for the daily physical activities (used the bus and walked, walked, or gardened, *P* < .001, or used the stairs, *P* = .04) (Figure 2). Adults aged 60 or younger who felt that they “cannot take the first step” to modify lifestyle behaviors at pretest decreased from 25.0% to 6.8% at posttest (*P* = .03) (Table 3). Adults who felt they were “taking action” to modify lifestyle behaviors increased from 31.5% to 63.0% (*P* < .001) for those 60 or younger and increased for those older than 60 from 30.0% to 45.0% (*P* < .001).

Daily Activity/Type of Exercise	Adults ≤60 y, %		*P* Value^a^	Adults >60 y, %		*P* Value^a^
	Pretest	Posttest		Pretest	Posttest	
**Daily activity**						
Used stairs	33.7	34.2	.22	60.7	73.3	.04
Used the bus and walked	41.2	29.9	.02	38.2	59.8	<.001
Walked	26.6	33.1	.02	56.9	82.5	<.001
Gardened	25.3	33.7	.03	20.7	36.5	<.001
**Type of exercise**						
Type A	19.8	52.5	.17	31.9	56.0	<.001
Type B	34.2	29.7	.04	54.1	71.6	<.001
Type C	30.6	65.0	<.001	39.8	76.1	<.001

^a^ *t*-test used to assess for significant differences of pretest to posttest responses within age group.

**Table 3:** Behavioral Change From Pretest (Baseline) to Posttest (12-Week Postintervention Follow-Up) Among Adult Participants in the *Salud para su Corazón* Mexican Pilot Study, 2009–2012

Response^a^	Adults ≤60 years (N = 279), %		*P* Value^a^	Adults >60 years (N = 173), %		*P* Value^b^
	Pretest	Posttest		Pretest	Posttest	
Not answered	12.0	2.5	.04	1.5	1.0	NA
Do not care	3.0	1.4	.09	1.5	1.0	.09
Cannot take the first step	25.0	6.8	.03	30.0	40.0	.13
I am planning to do it	12.5	12.3	.87	24.0	3.0	.09
I am taking action	31.5	63.0	<.001	30.0	45.0	<.001
I maintain a heart healthy lifestyle	16.0	14.0	.11	13.0	10.0	.24

Abbreviation: NA, not applicable (sample size too small [<10] for reliable testing).

a Question was, “Are you planning to modify your lifestyle behaviors?”

b χ^2^ test comparing differences of posttest to pretest responses within the same age group.

### Program feedback

We found no significant differences in the acceptance of the program among participants as evaluated by the pretest and 12-week follow-up surveys (data not shown). All adults 60 or younger and 98.8% adults older than 60 were satisfied with the educational materials provided during the sessions. All adults older than 60 and 97.8% of adults 60 or younger were satisfied with the instruction and guidance they received from the promotores during the sessions, and 97.8% adults 60 or younger and 93.8% adults older than 60 were satisfied with the encouragement and support they received from the promotores during the sessions.

## Discussion

The results of the *Salud para su Corazón* pilot study provide evidence that a cardiovascular disease prevention intervention, built around a model of community engagement and facilitated by promotores, is a promising practice-based program for improving cardiovascular knowledge and habits in a vulnerable, low-SES, urban adult population in Mexico City. The community accepted the challenge of working with the universities and incorporated self-identified needs that were associated with reduction of risk factors for CVD (21). The *Salud para su Corazón* model has demonstrated, and is further supported by the findings in this study, the potential to facilitate behavioral changes to improve cardiovascular health among participants, their families, and the promotores delivering the program (21). Finally, changes in cardiovascular disease risks observed in this study for blood glucose levels suggest that the promotores model can work successfully in community health promotion and disease prevention in Mexico.

The *Salud para su Corazón* pilot study in Mexico facilitated practical elements of learning to increase participants’ awareness of the risk factors for CVD that are prevalent in their community. The variety of topics in the curriculum were an interactive instructional mechanism that may have contributed to the regular attendance of participants to the educational sessions. Promotores were able to deliver the intervention and all sessions in the curriculum were completed by each group, with an average completion rate of 82% for the full 12-week intervention for all workshops. These results are similar to those of *Salud para su Corazón* programs in the United States among Latinos and Hispanics (17–20). This was the first program ever implemented in Mexico City, DF, by the newly trained group of promotores who were effective in enrolling community members into the *Salud para su Corazón* program.

The program has sustainable benefits. The train-the-trainer model of implementation was effectively realized, and 22 promotores remain in the community and continue to educate those in their social networks (21). Promotores working in pairs within the Mexican context was a process of program delivery well accepted by the promotores themselves and participants. Participants were receptive to the program and requested more sessions and more themes and wanted their classes conducted more frequently.

This study was conducted during a short period (12 weeks) for follow-up. We plan to extend follow-up at regular intervals (eg, 6 months and 1 year) to determine whether the changes were sustained or further improved (15). Evidence from other similar community-based cardiovascular health education interventions has shown significant improvements after 1 year or more (28–30).

Several limitations and challenges to program implementation were identified. Attrition rate of participants may have influenced the differences in the baseline and 12-week postintervention surveys. Promotores attempted to mitigate attrition through follow-up telephone calls and contacts. The lack of a control group was a limitation, and the findings may have been influenced by factors other than the intervention. Future activities would benefit from a control group or comparative effectiveness study of other health promotion activities to assess the most appropriate interventions within similar communities. The pilot study put significant time demands on university coordinators, and the logistics of visiting multiple sites in an urban area was challenging. Another challenge was the limited resources for program implementation, including a lack of funds to support promotores.

We found significant improvements in heart-healthy behaviors and actions to enhance positive lifestyle behaviors among participants and their families. The sustained capacity for health promotion in the community was established and can be used in future activities or programs. In conclusion, this study of the *Salud para su Corazón* program was effectively adapted and implemented in a vulnerable, low-SES population and has the potential to be replicated in other communities.
